# Influence of Timing on Surgical Outcomes for Acute Humeral Shaft Fractures

**DOI:** 10.1155/2021/8977630

**Published:** 2021-06-01

**Authors:** Ryogo Furuhata, Yusaku Kamata, Aki Kono, Yasuhiro Kiyota, Hideo Morioka

**Affiliations:** Department of Orthopedic Surgery, National Hospital Organization Tokyo Medical Center, 2-5-1, Higashigaoka, Meguro-ku, Tokyo 152-8902, Japan

## Abstract

Surgical treatment for humeral shaft fractures has been reported to yield satisfactory results; however, there may be complications, such as delayed bone union, nonunion, iatrogenic radial nerve injury, and infection. The risk factors for postoperative complications remain largely unknown. This study aimed to investigate the influence of timing of surgery on the incidence of postoperative complications of acute humeral shaft fractures. We retrospectively reviewed 43 patients who underwent osteosynthesis for acute humeral shaft fractures between 2006 and 2020. The patients were divided into early (21 patients) and delayed (22 patients) treatment groups based on the timing of the surgical intervention (within or after four days). Outcomes were the incidences of complications (delayed union, nonunion, iatrogenic radial nerve injury, and infection) and postoperative fracture gaps. We evaluated the outcomes using plain radiographs and clinical notes. In addition, we performed subgroup analyses on outcomes in a subgroup of patients who underwent intramedullary nailing and one who underwent plate fixation. The frequency of delayed union was significantly higher in the delayed group (*P*=0.046), and the postoperative fracture gap size was also significantly greater in the delayed group (*P*=0.007). The subgroup analyses demonstrated a significant association between the increased incidence of delayed union and delayed surgical interventions only in the intramedullary nailing subgroup (*P*=0.017). This study suggests that performing surgery within four days after acute humeral shaft fracture is recommended to reduce the occurrence of delayed union, particularly in cases requiring intramedullary nailing fixation.

## 1. Introduction

Surgical treatment of humeral shaft fractures has been reported to yield relatively satisfactory results compared to conservative treatment; however, there are complications, such as delayed bone union, nonunion, iatrogenic radial nerve injury, and infection [1–3]. To date, little has been reported on the factors affecting postoperative complications of humeral shaft fractures [3, 4]. Furthermore, the effects of the timing of surgery on the clinical outcomes for acute humeral shaft fractures remain unsolved.

This study aimed to investigate the influence of time from injury to surgery on the incidence of postoperative complications following acute humeral shaft fractures.

## 2. Materials and Methods

### 2.1. Patient Selection

This was a retrospective study in patients who underwent osteosynthesis for humeral shaft fractures between April 2006 and March 2020. All the patients were treated at a single general hospital, and surgeries were performed by eight surgeons. We included patients with acute humeral shaft fractures within three weeks of injury. Patients in whom the postoperative follow-up could not be performed adequately were excluded. Based on a previous study investigating the effects of timing of surgery for acute proximal humeral fractures [5], patients who met the above criteria were divided into patients who underwent surgery within four days after injury (early group) and those who were treated five or more days after injury (delayed group).

During the study period, 46 patients underwent osteosynthesis for acute humeral shaft fractures. Of these patients, two in the early group and one in the delayed group were excluded due to inadequate follow-up. Finally, 27 male and 16 female patients were included, and their mean age at the time of surgery was 45.2 ± 22.4 years (range: 15–89 years). Nineteen patients had a fracture in the right humerus, while 24 patients had a fracture in the left humerus. Fourteen patients (33%) were smokers. There were six patients with preoperative radial nerve palsy and one with an open fracture. The fracture types according to the Arbeitsgemeinschaft für Osteosynthesefragen (AO) classification [6] were type A in 27 patients (63%), B in 14 patients (33%), and C in two patients (4.7%). Four patients (9.3%) had proximal third fractures, and 15 patients (35%) had distal third fractures. Fixation procedures used were intramedullary nailing in 20 patients, plate fixation in 20 patients, fixation using Kirschner wire alone in one patient, and fixation using lag screws alone in two patients.

### 2.2. Outcomes

**Figure 1:**
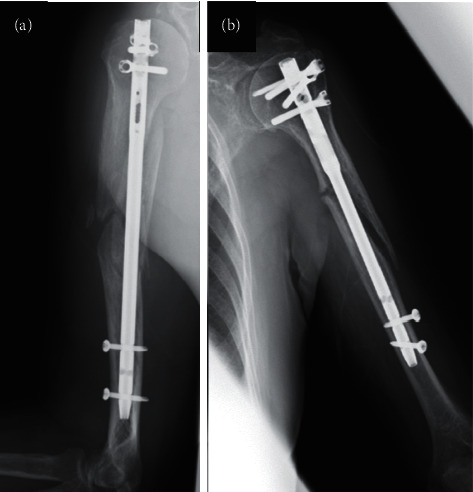
X-ray radiographs showing delayed bone union. A 70-year-old man sustained a humeral shaft fracture (AO type A1) and underwent an intramedullary nailing eight days after injury. A plain radiograph performed 26 weeks postoperation showed a visible fracture line (a). An 80-year-old woman sustained a humeral shaft fracture (AO type A2) and underwent an intramedullary nailing 11 days after injury. A plain radiograph performed 30 weeks postoperation revealed a visible fracture line and loosening distal screws (b).

The outcomes were complications (delayed union, nonunion, iatrogenic radial nerve injury, and infection) and postoperative fracture gaps. According to a previous report [3], union was defined as “bone bridging the fracture site across both cortices on radiographs taken in two planes,” and delayed union was defined as “union occurring after 26 weeks” (Figures 1(a) and 1(b)) A single examiner assessed postoperative plain radiographs. Iatrogenic radial nerve injuries were assessed based on medical records, and infections were assessed based on medical records and the use of antibiotics. Fracture gap immediately after surgery was measured on plain radiographs taken immediately after surgery as the shortest distance between the proximal and distal bone fragments, according to a previous report [7].

Subsequently, subgroup analyses were performed on outcomes in a subgroup of patients who underwent intramedullary nailing and a subgroup of patients who underwent plate fixation.

### 2.3. Statistical Analysis

All statistical analyses were conducted using SPSS software program (version 26.0, IBM, Armonk, NY, USA). We used the Mann–Whitney *U* test to compare the average of continuous values (age, time from injury to surgery, and postoperative fracture gap) and Fischer's exact test to compare the proportion of variables (sex, the side of injury, smoking, preoperative radial nerve injury, fracture type, fixation procedures, delayed union, nonunion, iatrogenic nerve injury, and infection) between the two groups. Continuous data are presented as mean ± standard deviation (SD). The threshold for significance was *P* < 0.05.

## 3. Results

Twenty-one patients who underwent surgery within four days after the injury were included in the early group, and 22 patients who underwent surgery five or more days after the injury were included in the delayed group. There was a significant difference in the time from injury to surgery between the early and delayed groups (*P* < 0.05); however, no significant differences were found in age, sex, injured side, percentage of smokers, the prevalence of preoperative radial nerve injury, fracture type distribution, or fixation procedures (Table 1).

In this study, eight (19%), two (4.7%), one (2.3%), and one (2.3%) patients experienced delayed union, nonunion, iatrogenic radial nerve injury, and infection, respectively. The frequency of delayed union was significantly higher in the delayed group (*P*=0.046). No significant differences between the two groups were noted in the rates of nonunion, iatrogenic radial injury, and infection. The postoperative fracture gap was significantly greater in the delayed group: 1.2 ± 1.7 mm in the early group and 3.5 ± 3.3 mm in the delayed group (*P*=0.007) (Table 2).

In addition, subgroup analyses were performed in a subgroup of patients who underwent plate fixation and a subgroup of patients who underwent intramedullary nailing. In the plate fixation subgroup, the incidence of delayed union was not significantly different between the early and delayed groups, whereas in the intramedullary nailing subgroup, delayed union occurred only in the delayed group at a significantly higher frequency (*P*=0.017). Similarly, the fracture gap size immediately after surgery was also significantly greater in the delayed group only in the intramedullary nailing subgroup (*P*=0.017) (Table 3).

## 4. Discussion

In this study, we investigated the influence of timing of surgery on the incidence of postoperative complications of acute humeral shaft fractures. As a result, we made two important clinical observations. First, surgical interventions for acute humeral shaft fractures five or more days after the injury were significantly associated with an increased incidence of delayed union. Second, this association between delayed surgical interventions and incidence of delayed union was observed only in a subgroup of patients who underwent intramedullary nailing.

First, the present study showed a significantly increased incidence of delayed union in the delayed group of patients with acute humeral shaft fractures compared with those in the early group. Although the difference in frequency was not significant, nonunion was observed only in the delayed group. The previously reported incidence rates of delayed union and nonunion after surgery for humeral shaft fractures are 33% [3] and 0–8.7% [1–3], respectively, and the results of this study were similar to these rates. According to a previous cohort study on humeral shaft fracture, initial surgical management without conservative treatment had a significantly higher union rate than delayed surgical management, which was initially treated using conservative management without achieving union and subsequently converted to surgical management [3]. These data suggest that delayed surgical intervention for humeral shaft fracture may be a risk factor for delayed union or nonunion; however, in acute fracture, the effects of time from injury to surgery on the union remain unclear. For acute proximal humeral fractures, surgery within five days after the injury has been recommended because postoperative complications significantly increased in patients who underwent surgery six or more days after the injury [5]; this result is similar to ours. Delayed surgical intervention is thought to complicate the anatomical fracture reduction and increase soft tissue dissection, which may result in a longer fracture union time [8, 9]. In this study, the fracture gap immediately after surgery is significantly greater in the delayed group, which supports this hypothesis that delayed surgical intervention makes the anatomical reduction difficult [8, 9]. The difficulty in an anatomical reduction in the delayed group may explain our result of a higher delayed union rate. Meanwhile, the incidence of iatrogenic radial nerve injuries did not differ significantly between the early and delayed groups in this study; this is analogous to a previously reported finding that delayed intervention for humeral shaft fractures did not increase the risk for radial nerve palsy [4]. The rate of postoperative infection in this study was 2.3%, which was similar to the rate of 3–5% reported in previous studies [10, 11]. In particular, postoperative deep wound infection is a serious complication that might be a risk factor for nonunion [12], and in this study, patients with postoperative deep wound infection had delayed bone union. However, the risk factors of postoperative infection have not been fully elucidated. The incidence of infection after plate fixation has been reported to be four times higher than after intramedullary fixation [13], which suggest that plate fixation can be a risk factor for postoperative infection; however, in this study, the only patient who developed postoperative infection underwent intramedullary nail fixation. In addition, the results of this study demonstrated that the delayed surgical intervention had no significant effect on the rate of postoperative infection.

Second, in this study, all except one patient with delayed union or nonunion were in the intramedullary nailing group, and the subgroup analyses demonstrated a significant association between the increased incidence of delayed union and delayed surgical intervention only in the intramedullary nailing subgroup, and not in the plate fixation subgroup. With respect to bone union, many reports have shown no significant differences between the patients who underwent intramedullary nailing and the patients who underwent plate fixation [3, 10, 14, 15]. However, one report showed that bone union tended to be earlier in the plate fixation group [11]; thus, the issue remains controversial. The results of the present study suggest that the incidence of delayed union may be affected by the time from injury to surgery only in patients who underwent intramedullary nailing. In addition, a significant difference in the postoperative fracture gap between the early and delayed groups was also observed only in the intramedullary nailing subgroup. The greater fracture gap size of delayed surgical intervention in the intramedullary nailing subgroup is presumably because closed reduction without opening the fracture site was performed in most patients in this subgroup; however, the fracture site was directly opened and reduced/fixed in most patients in the plate fixation subgroup. Given a previous report on conservative treatment for humeral shaft fractures identifying the magnitude of the fracture gap as a risk factor for subsequent fracture instability and nonunion [7], our findings suggest that the residual fracture gaps immediately after surgery could have affected the incidence of delayed union in patients who underwent the delayed treatment using intramedullary nails. In this study, closed reduction in most patients who underwent intramedullary nailing may have caused the inadequate axis loading at the fracture sites in the intramedullary nailing subgroup.

This study has three major limitations. First, the sample size in this study was small; only 43 cases were included in the study. If a greater number of patients were included, significant differences between the early and delayed groups might have been observed in the incidence rates of postoperative complications. Second, because this was an observational study, biases from unobserved differences may have affected the results. For example, the surgeries in this study were performed by eight surgeons; however, the effects of competencies of the surgeons or surgical assistants were not evaluated. In contrast, female sex, smoking, and proximal third fractures have been reported to be risk factors for fracture instability in a report on conservative treatment for humeral shaft fractures [7]; the frequencies of these risk factors were not changed between the early and delayed groups in this study. Finally, questionnaire surveys on pain, range of motion, and function were not included in this study; thus, more objective functional outcome scoring was not feasible.

## 5. Conclusions

This study provides new information on the timing of surgery for acute humeral fractures. Delayed surgical intervention five or more days after the injury was significantly associated with postoperative delayed union of humeral shaft fractures. Moreover, the subgroup analysis in this study suggests that plate fixation is more advantageous than intramedullary nailing in patients who fail to undergo surgical treatment within four days after injury, considering bone union.

## Figures and Tables

**Table 1:** Patient demographics.

	Early group (*n* = 21)	Delayed group (*n* = 22)	*P* value
Time from injury to surgery (days)	2.5 ± 1.3	7.0 ± 1.8	<0.001^*∗*^
Age (years)	43.1 ± 21.8	51.8 ± 22.2	0.21
Male/female	12/9	15/7	0.54
Side of injury, right/left	8/13	11/11	0.54
Smoker/nonsmoker	6/15	8/14	0.75
Preoperative radial nerve injury	3	3	1.0
AO type A	12	15	0.54
AO type B	8	6	0.52
AO type C	1	1	1.0
Proximal 1/3	1	3	0.61
Middle 1/3	11	12	1.0
Distal 1/3	9	6	0.35
Intramedullary nailing	7	13	0.13
Plate fixation	12	8	0.23
Kirschner wire or lag screw	2	1	0.61

^*∗*^ *P* < 0.001. AO: Arbeitsgemeinschaft für Osteosynthesefragen.

**Table 2:** Comparison of incidence of postoperative complications and postoperative fracture gap (early group vs. delayed group).

	Early group (*n* = 21)	Delayed group (*n* = 22)	*P* value
Delayed union	1 (4.8%)	7 (32%)	0.046^*∗*^
Nonunion	0 (0%)	2 (9.1%)	0.49
Iatrogenic radial nerve injury	1 (4.8%)	0 (0%)	0.49
Infection	0 (0%)	1 (4.5%)	1.0
Postoperative fracture gap	1.2 ± 1.7	3.5 ± 3.3	0.007^*∗*^

^*∗*^ *P* < 0.05.

**Table 3:** Subgroup analysis of the incidence of postoperative complications and postoperative fracture gap in intramedullary nailing group or plate fixation group.

Intramedullary nailing	Early group (*n* = 7)	Delayed group (*n* = 13)	*P* value
Delayed union	0 (0%)	7 (54%)	0.017^*∗*^
Nonunion	0 (0%)	2 (15%)	0.51
Postoperative fracture gap (mm)	1.9 ± 1.4	4.8 ± 3.3	0.017^*∗*^

Plate fixation	Early group (*n* = 12)	Delayed group (*n* = 8)	*P* value

Delayed union	1 (8.3%)	0 (0%)	1.0
Nonunion	0 (0%)	0 (0%)	—
Postoperative fracture gap (mm)	0.5 ± 0.7	1.9 ± 2.4	0.172

^*∗*^ *P* < 0.05.

## Data Availability

The datasets used and analyzed during the current study are available from the corresponding author on request.
